# Life Expectancy Gain of Implementing the Nordic Nutrition Recommendations 2023: Modeling From 8 Nordic and Baltic Countries

**DOI:** 10.1016/j.cdnut.2024.104540

**Published:** 2024-12-28

**Authors:** Elaheh Javadi Arjmand, Erik K Arnesen, Øystein Ariansen Haaland, Jan-Magnus Økland, Katherine M Livingstone, John C Mathers, Carlos Celis-Morales, Kjell Arne Johansson, Lars T Fadnes

**Affiliations:** 1 Department of Global Public Health and Primary Care, University of Bergen, Bergen, Norway; 2 Bergen Addiction Research, Department of Addiction Medicine, Haukeland University Hospital, Bergen, Norway; 3 Department of Nutrition, Institute of Basic Medical Sciences, Faculty of Medicine, University of Oslo, Oslo, Norway; 4 Bergen Centre for Ethics and Priority Setting, Department of Global Public Health and Primary Care, University of Bergen, Bergen, Norway; 5 Institute for Physical Activity and Nutrition (IPAN), School of Exercise and Nutrition Sciences, Deakin University, Geelong, Victoria, Australia; 6 Human Nutrition & Exercise Research Centre, Centre for Healthier Lives, Population Health Sciences Institute, Newcastle University, Newcastle upon Tyne, United Kingdom; 7 Education Physical Activity and Health Research Unit, University Católica del Maule, Talca, Chile; 8 School of Cardiovascular and Metabolic Health, University of Glasgow, Glasgow, United Kingdom

**Keywords:** life expectancy, longevity, Nordic Nutrition Recommendations, dietary recommendations, Nordic countries, Baltic countries

## Abstract

**Background:**

Dietary guidelines play a key role in promoting health and preventing chronic diseases. The Nordic Nutrition Recommendations (NNR) 2023 provide updated recommendations for healthy eating relevant for the Nordic and Baltic countries, but the potential benefits have yet to be quantified.

**Objectives:**

This study aimed to project the population health benefits, specifically, potential gains in life expectancy in Nordic and Baltic countries resulting from long-term dietary changes from current dietary patterns within each country to NNR2023.

**Methods:**

For this population-based mathematical model, using the Food4HealthyLife 2.0 calculator, data were obtained from meta-analyses on associations between each food group and mortality, and background mortality data were derived from the Global Burden of Disease study. Standard life-table methods were used, accounting for the correlation between 14 food groups and the anticipated time delay between dietary changes and health effects.

**Results:**

For 40-y-old females and males, projected life expectancy gains were from 1.8 and 2.1 y in Finland to 3.4 and 4.1 y, respectively, in Lithuania, changing to feasible NNR2023. Correspondingly, when changing to full-potential NNR2023, gains ranged from 4.4 and 5.0 y in Finland to 6.1 and 7.3 y, respectively, in Lithuania. The largest gains in life expectancy were linked to consuming more legumes (18%), nuts (17%), whole grains (12%), and less processed meat (14%) and added sugars (13%).

**Conclusions:**

Adopting dietary patterns in line with the NNR2023 is associated with considerable gains in life expectancy in the Nordic and Baltic countries. The study contributes to the evidence base to support policy measures to achieve NNR2023.

## Introduction

Dietary guidelines are an important tool for promoting a healthy lifestyle [1]. The WHO has encouraged the creation of national food-based dietary guidelines that adhere to sustainable healthy diets, taking into account each nation’s unique environmental, social, cultural, and economic circumstances [2]. For ∼40 y, the Nordic Nutrition Recommendations (NNRs) have served as the scientific foundation for national dietary reference values for nutrients as well as for the food-based dietary guidelines in the Nordic regions (Denmark, Finland, Iceland, Norway, and Sweden), and now also for countries in the Baltic region (Estonia, Latvia, and Lithuania) [3]. In 2019, between 11% and 25% of all deaths were caused by dietary risk factors in these countries [4]. The NNRs are among the most scientifically comprehensive dietary guidelines with specific food-based recommendations, and the final version (NNR2023) was published in June 2023 after a 5-y revision process involving several hundred leading experts [[5], [6], [7]]. The intended target group for the NNRs is the general population within the region. Food-based recommendations were based on epidemiologic evidence, clinical studies, and mechanistic studies for prospective associations with health outcomes relevant to the Nordic and Baltic populations and on the food groups’ contributions to nutrient intakes. Previously, the NNRs have addressed the beneficial and adverse effects of each food group on health, based on patterns of food consumption in the countries [3,8,9]. Moreover, in NNR2023, the impact of food consumption on planetary health was also assessed although the recommendations were primarily based on health outcomes [3].

Modeling life expectancy (LE) gains based on improvements in dietary patterns can provide policy-relevant evidence to underpin dietary guidelines [10,11]. The Global Burden of Disease results tool, Global Health Data Exchange (GHDx), provides estimates of overall nutritional risk factors but does not provide projections of gains in health following the implementation of guidelines [4]. Models, such as the Food4HealthyLife, have illustrated how modifications in food choices can be associated with substantial changes in age-specific LE [11]. Age-specific LE is a population measure of health status and estimates how much longer individuals at a certain age are expected to live. LE at birth is a common summary measure of a population’s health [12]. At the population level, changes toward diets with higher intakes of whole grains, fruits and vegetables, legumes, and nuts and lower intakes of red meat, processed meat, and added sugars are expected to improve LE [11,13]. Consequently, implementation of the NNR2023 is likely to have health benefits for people in the Nordic and Baltic countries, but the potential gains in LE have not been estimated.

In this article, we used the Food4HealthyLife model to project the gain in sex-specific and age-specific LE of adhering to the food-based recommendations in the NNR2023 in each of the Nordic and Baltic countries.

## Methods

**FIGURE 1:**
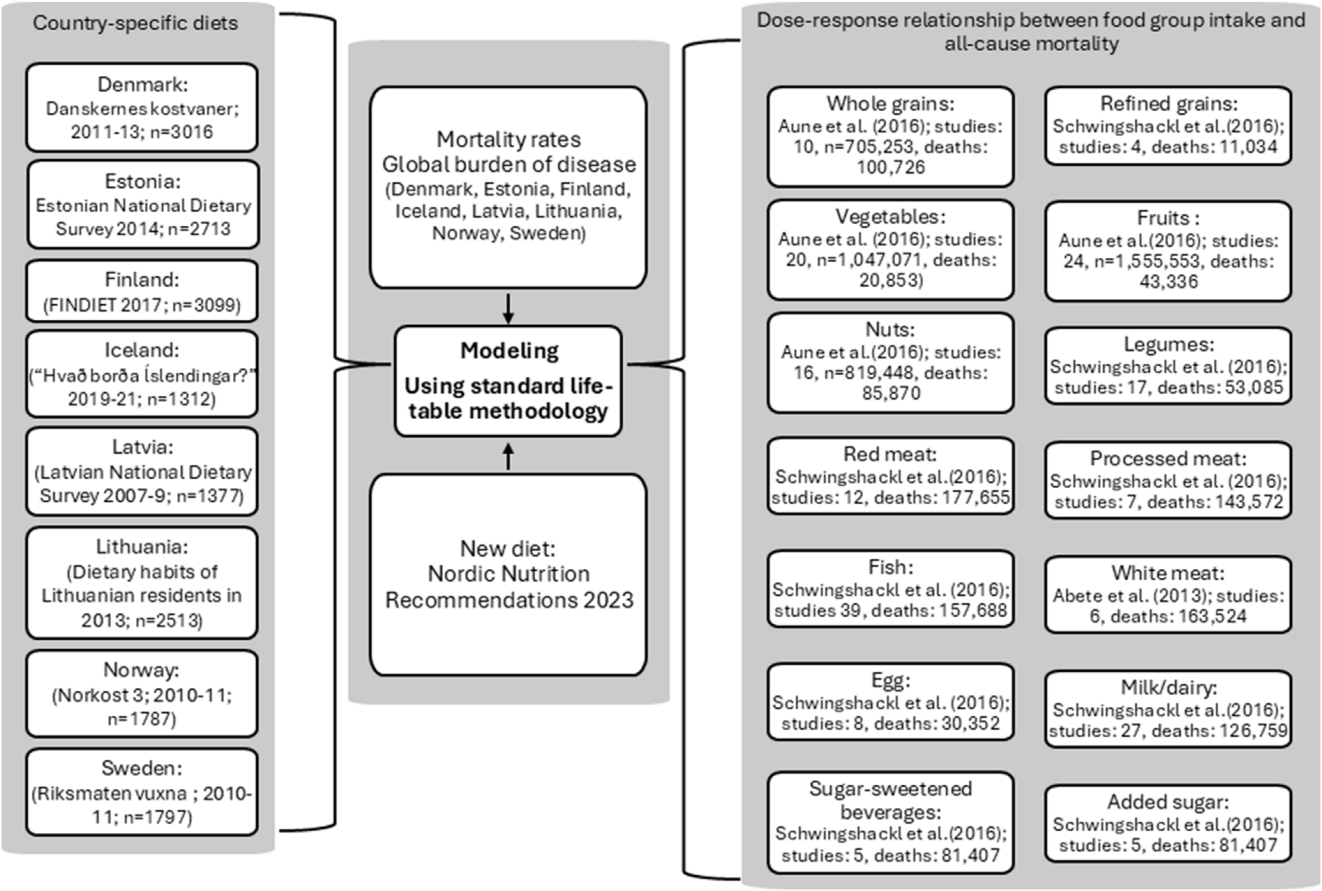
Participant and data flow chart summarizing input data with the number of studies, participants, and death outcomes for each food group exposure as well as country-specific diet, new diet, and background mortality data.

We will first provide a brief description of the Food4HealthyLife model, which is described in more detail in Johansson et al. [14], Fadnes et al. [11], and Supplemental Texts 1 and 2. A summary of the studies used for the model is described in Figure 1. We used the TRIPOD checklist while writing our report [15].

### Conceptual framework

The Food4HealthyLife model uses standard life-table methodology to calculate gains or losses in age-specific, sex-specific, and country-specific LE based on sustained dietary changes. The key inputs were current (baseline) age-specific mortality rates (i.e. before dietary changes), country-specific dietary patterns at baseline, and estimated effect on all-cause mortality following the dietary changes. Inputs on country-specific, age-specific, and sex-specific mortality rates were obtained from the 2019 Global Burden of Diseases and Injuries study [4]. Meta-analyses provided input on the dose–response relationship between the intake of each food group and all-cause mortality (Supplemental Figure 1). As baseline input on dietary patterns, the model used sex-specific data on dietary patterns for each of the Nordic and Baltic countries (Table 1). After a hypothetical individual changed their diet from the current diet in a chosen country, their LE shifted from LE_baseline_ (age, sex, and location) to LE_new_ (age, sex, and location). Gains or losses in LE were estimated by the difference between the 2 and are presented with uncertainty intervals (UIs).

**TABLE 1:** Current Food Consumption in Nordic and Baltic Countries, as well as NNRs proposed diet

	Denmark		Estonia		Finland		Iceland		Latvia		Lithuania		Norway		Sweden		NNR (female and males)	
	Female	Male	Female	Male	Female	Male	Female	Male	Female	Male	Female	Male	Female	Male	Female	Male	Feasible	Full-potential
Whole grain products^1^	93	130	86	79	157	210	180	197	86	79	75	71	160	203	130	153	300	300
Vegetables including potatoes	271	308	215	269	239	250	155	165	262	249	182	189	205	220	243	290	350	500
Fruits	212	166	235	182	189	135	99	94	165	127	170	150	189	179	147	105	250	400
Nuts	5	3	4	4	9	7	5	5	8	6	0	0	4	4	5	4	20	30
Legumes^2^	5	3	5	7	14	12	15	13	17	18	0	0	5	8	30	30	50	150
Fish	34	40	23	29	27	36	39	52	26	36	27	29	52	74	37	43	40	65
Egg	23	26	20	26	24	24	17	22	31	40	23	34	23	28	14	14	50	25
Milk and dairy^3^	581	690	365	444	611	740	503	629	185	224	124	140	564	729	415	455	500	350
Refined grains (dry)	57	75	56	67	64	86	37	46	57	77	59	77	54	82	50	66	Unchanged	Unchanged
Red meat	65	103	36	61	42	79	41	65	37	63	30	49	44	72	45	74	40	0
Processed meat	34	69	14	24	29	59	21	40	63	111	51	86	36	58	23	40	10	0
White meat	24	29	17	23	36	43	29	41	36	50	29	39	20	26	20	23	Unchanged	Unchanged
Sugar-sweetened beverages	181	258	40	82	62	117	75	114	61	128	36	74	89	171	70	102	0	0
Added sugars	43	56	53	53	39	43	36	43	80	80	80	80	35	48	44	53	25	0
Calculated energy	7458	9520	5756	6711	7526	9504	5910	7238	6870	8328	5938	7116	6811	9044	5934	6921	8036	7607
Uncategorized (%)	31	8	51	40	31	10	49	34	39	23	49	36	39	15	49	38	26	31

Intake from each food group in grams per day, estimated calculated energy (kJ/d), and estimated percentage from noncategorized intake groups are presented.

1 Whole grain products intakes are presented in fresh weight; dry weight was converted to fresh weight by a 3.33 conversion ratio.

2 Legumes intakes are presented in prepared weight; dry weight was converted by a 2.5 conversion ratio.

3 Milk and dairy intake include milk, yogurt, and cheese (cheese intake was converted to milk by 7.5 conversion ratio).

The model also includes a parameter that accounts for the fact that the mortality reduction from each food group may not be independent (i.e. the effect of changing the intake of 1 food group may modulate the effect of changing the intake of another food group), and another parameter that accounts for the fact that it may take many years before the full impact of the dietary changes on mortality is evident. More details about the conceptual framework are presented in Supplemental Text 1.

### Country-specific diets

To account for the diversity of diets across individuals and contexts, we used data from the most recent national surveys to estimate current dietary patterns in Nordic and Baltic countries also presented in an article linked to the NNR2023 [5]. The characteristics and references to the surveys used to define a current diet for each country are provided in Supplemental Text 2. Table 1 shows the average intake of each of the 14 food groups among adults in each of these countries, that is, whole grain products, vegetables (including potatoes), fruits, nuts, legumes, fish, eggs, milk/dairy, refined grains, red meat, processed meat, white meat, sugar-sweetened beverages, and added sugars. Several countries reported consumption of food groups that do not fall into any of the dietary groups specified in Table 1. Therefore, if the calculated total energy from intake of each of the food categories was below the estimated daily energy intake, we assumed the remaining energy was derived from such uncategorized foods. As the number of uncategorized food groups increases, the width of the UIs increases. The estimation of energy intake is derived from energy expenditure projection equations proposed by Heymsfield et al. [16].

### Mortality impact

Recent and extensive meta-analyses were used to establish dose–response relationships between the consumption of food groups and all-cause mortality (Supplemental Text 1). The assessment of the overall quality of evidence was conducted by using NutriGrade scores for studies included in each distinct food category [17], with weighting for absolute contribution to the change in LE.

### Target diet and online calculator

The NNR2023 food-based recommendations were retrieved from the published report [18]. Most recommendations were provided as a range, for example, “consume a variety of vegetables, fruits, and berries, 500–800 grams, or more, per day in total” and in some cases included only qualitative advice. Thus, we present 2 interpretations of the advice on the daily intake of food groups: One closer to the current dietary patterns (feasible) and one closer to the levels associated with the lowest mortality (full-potential) (Supplemental Text 1). To estimate the impact of each dietary modification, we used the R package Shiny to develop a web application (http://v2.food4healthylife.org/) [19,20]. In this study, we provided the projected LE gain for 40-y-old adults as an example of age-specific LE and compared the same age group between males and females and among countries. Plots are generated via the R package Highcharter [21].

## Results

**FIGURE 2:**
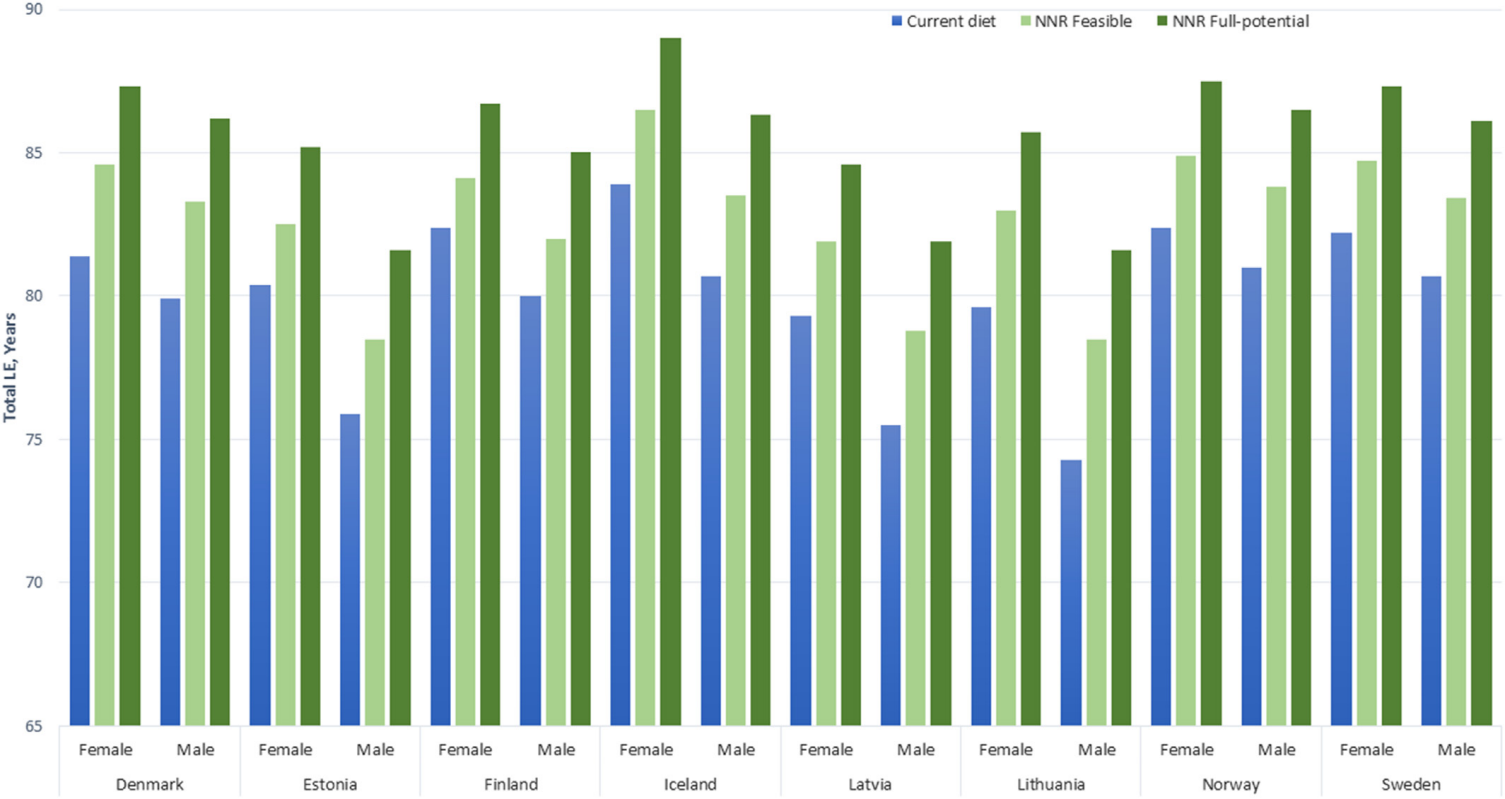
Estimated life expectancy (LE) from birth in Nordic and Baltic countries among females and males with current national diet and dietary patterns in line with the Nordic Nutrition Recommendations 2023: feasible (close to current diets) or full-potential.

The projected LE_birth_ with a current national diet ranged from 79.3 (Latvia) to 83.9 y (Iceland) for females and from 74.3 (Lithuania) to 81 y (Norway) for males (Figure 2). The current diets varied between countries. For example, the intake of whole grains was lower in the Baltic countries than that in the Nordic countries (Table 1). In all countries, the intake of nuts and legumes was generally low, whereas the intake of fruits and vegetables was mostly above half of the lower recommended intake range. The intake of red meat and processed meat was higher than the recommended intake in most countries. Finally, sugar-sweetened beverages intake was higher in Nordic countries than that in Baltic countries and higher in males than that in females.

**FIGURE 3:**
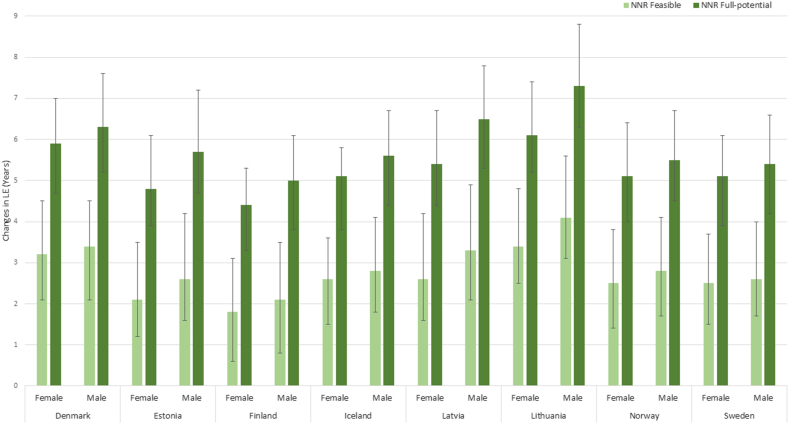
Life expectancy (LE) gain (years) in Nordic and Baltic countries among 40-y-old females and males when changing from their current national diet to dietary patterns in line with the Nordic Nutrition Recommendations 2023: feasible (close to current diets) or full-potential. Estimates are presented with uncertainty intervals.

For a 40-y-old reference person in Nordic and Baltic countries, the projected LE gained from a sustained change from the current dietary pattern in the respective country to the recommendations of NNR2023 is presented in Figure 3. For the feasible NNR2023, LE gain ranged from 1.8 y (UI: 0.6, 3.1 y) in Finland to 3.4 y (UI: 2.5, 4.8 y) in Lithuania among females and from 2.1 y (UI: 0.8, 3.5 y) in Finland to 4.1 y (UI: 3.1, 5.6 y) in Lithuania among males. For the full-potential NNR2023, LE gain ranged from 4.4 y (UI: 3.3, 5.3 y) in Finland to 6.1 y (UI: 5.2, 7.4 y) in Lithuania among females and from 5.0 y (UI: 3.8, 6.1 y) in Finland to 7.3 y (UI: 6.3, 8.8 y) in Lithuania among males. The resulting LE_birth_ for the feasible NNR2023 ranged from 81.9 y (Latvia) to 86.5 y (Iceland) for females and from 78.5 y (Estonia) to 83.8 y (Norway) for males. For the full-potential NNR2023, the resulting LE_birth_ ranged from 84.6 y (Latvia) to 89.0 y (Iceland) for females and from 81.6 y (Estonia) to 86.5 y (Norway) (Figure 2).

**FIGURE 4:**
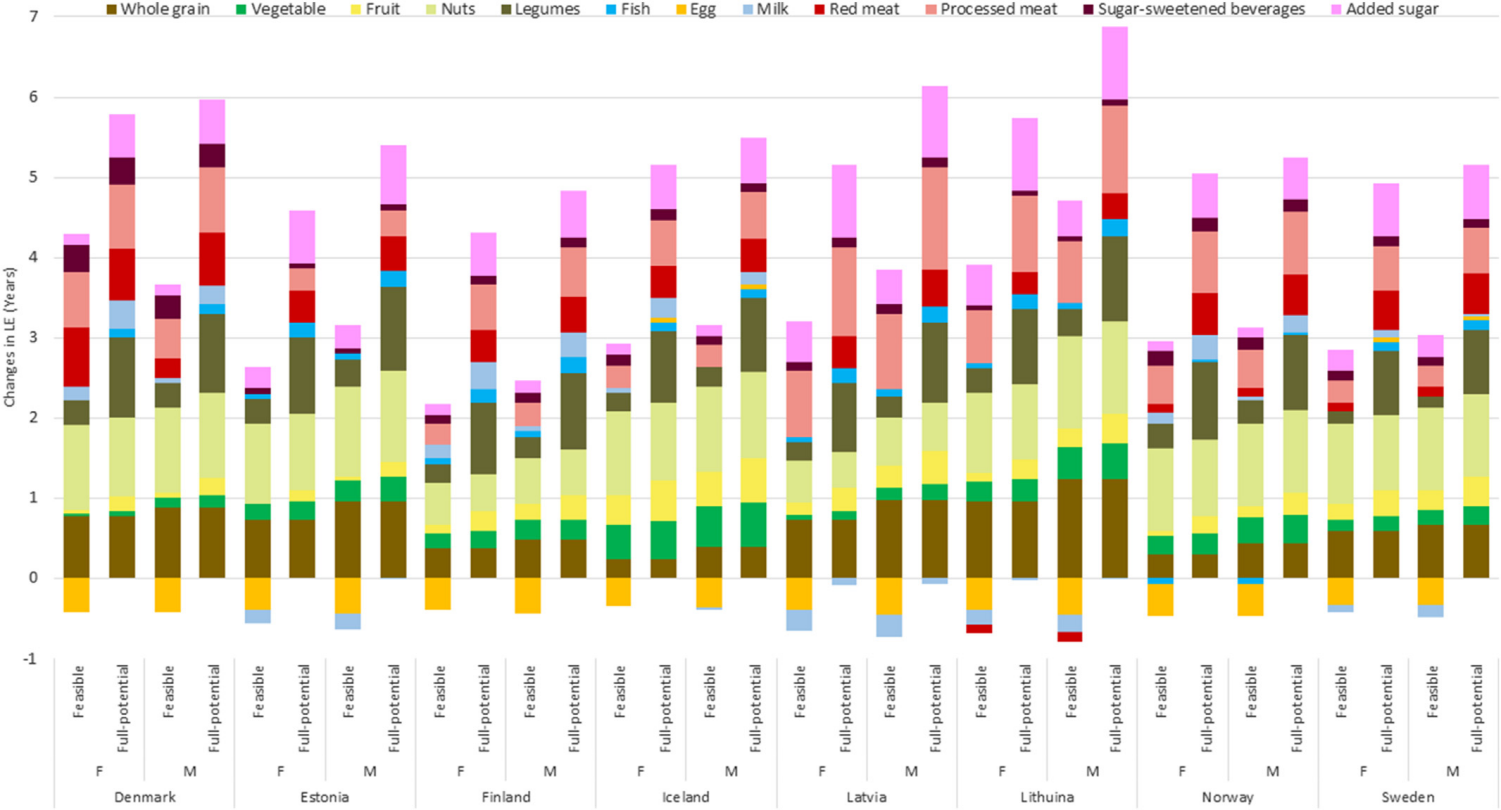
Estimated changes in food-specific life expectancy (LE) in years from sustained change from current diets in the Nordic and Baltic countries to feasible- and full-potential NNR2023 among 40-y-old females (F) and males (M). ∗Refined grains and white meat had no contributions since the new diet includes no change in the intake.

**FIGURE 5:**
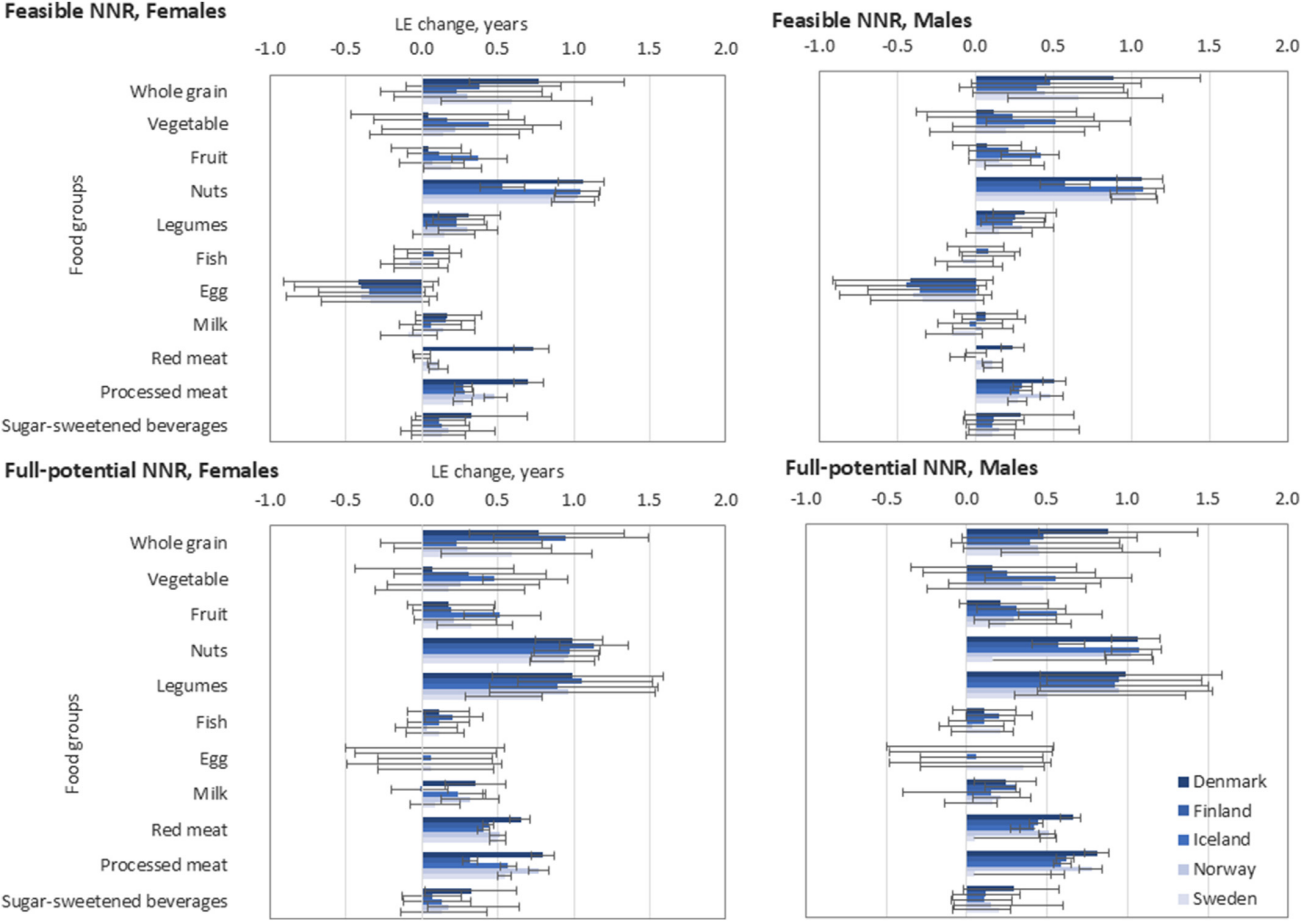
Estimated changes in food-specific life expectancy in years from sustained change from current diets in the Nordic and Baltic countries to feasible (top) and full-potential (bottom) interpretation of the NNR2023 among 40-y-old females (left) and males (right). Presented with uncertainty intervals. *∗Refined grains and white meat had no contributions since the new diet includes no change in the intake*.

The contributions made by changes in intake of each of the food groups to LE change are presented in Supplemental Tables 1 and 2 and FIGURE 4, FIGURE 5. The largest LE gain would be achieved by eating more legumes (18%), nuts (17%), whole grains (12%), and less processed meat (14%) and added sugars (13%) (Supplemental Table 3). For a 40-y-old female in Denmark with a sustained change to the feasible NNR2023, the largest gains in LE are from increasing the intake of nuts (1.1 y; UI: 0.9, 1.2 y), whole grains (0.8 y; UI: 0.3, 1.3 y), and legumes (0.3 y; UI: 0.1, 0.5 y) and decreasing the intake of processed meat (0.7 y; UI: 0.6, 0.8 y) and red meat (0.7 y; UI: 0.6, 0.8 y). Correspondingly, for a 40-y-old male in Lithuania with a sustained change to the full-potential NNR2023, the most impactful changes would be eating more whole grains (1.2 y; UI: 0.8, 1.8 y), nuts (1.2 y; UI: 1.0, 1.3 y) and legumes (1.1 y; UI: 0.5, 1.7 y) and eating less processed meat (1.1 y; UI: 1.0, 1.2 y) and added sugars (0.9 y; UI: 0.5, 1.4 y). For some of the food groups such as eggs, clear gains in LE from adaptation of NNR2023 were unlikely (Figure 5). For the feasible NNR2023, the changes in LE following the change in intake of eggs were negative, with an UI ranging from −1.0 to 0.11 y.

## Discussion

This study shows a large, projected gain in LE from long-term shifts in the current dietary patterns in Nordic and Baltic countries to the NNR2023. For 40-y-old adults, adhering to NNR2023 was associated with gains of ≤4 y for the feasible intrepretation (closer to current national diets) and gains of ≤7 y when interpreted toward full-potential ranges of NNR2023 . The LE gain for males was higher than that for females because females generally had healthier diets than males initially.

Increasing the intake of whole grains, nuts, and legumes and decreasing the intake of red and processed meat as well as added sugars were estimated to contribute most to the gains in LE. For nuts and legumes, the current intakes in the Nordic and Baltic countries were particularly low. Since current intakes of meat products are higher than the maximum recommended amount in most countries [5], replacing meat products with plant-based protein sources such as legumes would contribute substantially to the gain in LE. In contrast, although a higher intake of fruits and vegetables is associated with reduced mortality [22,23], changes in intakes of fruits and vegetables are associated with slightly lower gains as the intakes are already closer to the recommendations in most Nordic and Baltic countries than for nuts, legumes, and whole grains [5]. Furthermore, when adopting a feasible NNR2023, it becomes evident that certain food groups, notably eggs and milk/dairy, had negative contributions to LE in some countries. This observation aligns with our expectations, given that the full-potential interpretation tends toward a diet with better health outcomes. It is worth mentioning that the contributions of food groups to LE gain can differ in other settings, for example, a recent study in the United Kingdom using the same model, reported the largest gain from consuming more whole grains, nuts, and fruits and less sugar-sweetened beverages and processed meat [10].

The findings of this study have implications for those developing and implementing health-related policies, for clinicians, and for citizens. The projected LE gains not only support adherence to feasible and full-potential interpretations of NNR2023 but also demonstrated the public health implications of implementing sustained small dietary changes across the Nordic and Baltic counties. Moreover, understanding the impact of food choices on age-specific LE is valuable for policymakers when setting priority actions to meet the sustainable development goal 3.4 of reducing premature mortality from noncommunicable diseases by 30% by 2030. Our findings are also relevant for clinicians when treating and advising patients on dietary changes to optimize health and for citizens when prioritizing different food choices in everyday life.

There are certain limitations inherent in our study. First, the quantitative food-based recommendations in NNR2023 were derived from convincing or probable causal dose–response relationships in qualified systematic reviews, some of which had outcomes other than mortality, and thus did not always overlap with the systematic reviews included in this study [3]. The systematic reviews and meta-analyses used in this study may not necessarily adhere to the strict inclusion criteria in NNR2023. Second, the meta-analyses used in our model included observational studies only. This data source often exhibits heterogeneity, where the total estimates may not fully account for the variations in settings and the varying degrees of correction for potential confounding factors. However, meta-analyses frequently provide the most comprehensive and unbiased evidence available because they typically account for several of the most important confounding factors. Studies that investigate associations between food groups and mortality before and after adjustment for intake of other food groups in addition to other confounders, often demonstrate minimal alterations in the estimates of mortality risk [[24], [25], [26]]. Studies using alternative experimental designs such as randomized control trials could demonstrate different results, but such studies focusing on dose–response relationships between food groups and all-cause mortality are rare. Updated meta-analyses, published since our model was developed, may provide greater precision in projections of LE gains. Third, our approach is limited for individual use because the projections do not account for previous or current morbidity, nor do they incorporate other risk factors such as genetic susceptibility, advancements in medical therapies, or lifestyle modifications [27]. Hence, the methodology used in this study is not intended for personalized projection of life-years gained, but rather for estimating gains at the population level based on specific assumptions of the model. In addition, the gain in LE could be used as a tool to indicate the likely health benefits of the recommendations and to act as a motivation for individuals to change their diet. According to meta-analyses, adhering to optimum diets is likely to have a protective impact on the risk of obesity [28], and this might mediate some of the effects of dietary change on LE. A further limitation is that the current food consumption underlying our estimations are self-reported data from dietary surveys (most using 24-h recall methods), which are prone to information bias, some of which were conducted >1 decade ago. However, the results are broadly similar to the latest Global Burden of Disease analysis, which used various data sources to impute dietary exposures, and found that low whole grain and fruit consumption and high red and processed meat consumption were among the largest attributable dietary risk factors for deaths in the Nordic and Baltic countries [29]. Furthermore, relying on average intakes from dietary surveys does not capture variability in consumption patterns across individuals within populations. The definitions of the food groups may also have differed between countries. The reported intakes of fats/oils in most countries were incompatible with the background data that we have used in our model; thus, we excluded the fats/oil intake from the current diet, and it was estimated with the uncategorized food group. Our approach also has strengths. The new NNR recommendations used in this study provide one of the most comprehensive dietary guidelines to date and consider the health effects as well as sustainability [18,30]. Our study used a model that incorporates extensive meta-analyses containing dose–response data that adjust for correlations between food group consumption and the risk of all-cause death. Although our model is based on all-cause mortality and does not account for nonfatal outcomes, existing literature indicates that diet greatly affects the risk of common noncommunicable diseases and health-related quality of life [23,28,[31], [32], [33], [34]]. Further, dietary risk factors for several morbidities have a large degree of overlap with associations with all-cause mortality [23,31,32]. Additionally, we established a methodology that incorporates the likely time delay in achieving the health effects following a change in dietary pattern. The quality of the evidence for the various food groups with health outcomes spanned from very low for eggs and white meat to high for whole grains, with the majority falling within the moderate range [17], and the total level of evidence was assessed as moderate.

In conclusion, changing from a current diet to the dietary pattern recommended in NNR2023 is projected to produce substantial gains in LE in all Nordic and Baltic countries. For 40-y-old adults, this LE gain could be in the range of 4–7 y. The study contributes to the evidence base to support the development and implementation of policy measures designed to achieve implementation of the NNR2023.

## Author contributions

The authors’ responsibilities were as follows – LTF, J-MØ, ØAH, KAJ: have been involved in the design of the study; EJA, LTF, EKA, J-MØ: have been involved in the analysis of the study; LTF: is the principal investigator; EJA: wrote the first draft of the paper; and all authors: participated in the interpretation of the data, reviewed the manuscript for intellectual content, read and approved the final version of the manuscript.

## Data availability

Data used in this study are available through various online sources (see Supplementary Material and references to a range of sources).

## Funding

This work was supported by the Trond Mohn Foundation, grant number TMS2019TMT02. The hosting for the computations was performed on the Norwegian Research and Education Cloud (NREC), using resources provided by the University of Bergen and the University of Oslo (http://www.nrec.no/). The authors were funded by their respective institutions. The funders had no role in the study design, analysis, decision to publish, or preparation of the manuscript. EJA was funded through Helse Vest on the ATLAS4LAR project. KML is supported by a National Health and Medical Research Council Emerging Leadership Fellowship (APP1173803).

## Conflict of interest

The authors report no conflicts of interest.
