# Supplementary material for: Life Expectancy Gain of Implementing the Nordic Nutrition Recommendations 2023: Modeling From 8 Nordic and Baltic Countries
Source: Curr Dev Nutr. 2024 Dec 28;9(2):104540. doi: 10.1016/j.cdnut.2024.104540 (PMC11786883; doi:10.1016/j.cdnut.2024.104540)
Supplement: Multimedia component 1 [file mmc1.docx]

**Supplementary Text 1: Methodological details**

**Extraction of background mortality data**

Data on background mortality from 2019 for specific countries and regions were obtained from the freely available GBD cause of death database (1). There were data available for year 2020 and 2021, but due to COVID-19 we opted to use mortality rates in the pre-COVID-19 era extracted from GBD 2019 (published in 2020). We extracted data for all the included countries. Region-specific estimates on total mortality rates in 5-year age groups were also available from GBD. These were converted to single-year age specific mortality rates in our model.

**Extraction from meta-analyses**

Recent meta-analyses provided dose-response data on the impact of various food groups on mortality for the following food groups: whole grains, fruits, vegetables, nuts, legumes, fish, eggs, milk/dairy, refined grains, red meat, processed meat, sugar-sweetened beverages and foods, and added oils (2-7). Other food groups were not considered. When more than one meta-analysis was available, we opted for the most comprehensive (usually the latest) with dose-response relationship data unless later less comprehensive meta-analyses argued well for excluding the studies. Most of the studies were adjusted for factors such as smoking, exercise, body mass index, age, and sex, and many also adjusted for intake of other food groups. Each of the food groups were considered as individual protective or risk factors. However, due to some correlation between the food groups with some residual overlap between the relative risks, we used a model adjustment (“penalty”) that 25% overlap between the relative risk estimates.

For several of the food groups, more than one meta-analysis is available. For red and processed meats, a more recent meta-analysis from 2019 than the one used in our estimates has been published (8). However, this did not present dose-response data for red and processed meats separately, and the supplementary data for these groups combined indicated similar results as for the meta-analysis by Schwingshackl et *al*. It is worthy to note that meta-analyses indicate worse outcomes on life expectancy from processed meat than non-processed red meat when compared by weight, but if the consumption of unprocessed red meat consumption is twice as high as for processed meat the total effect is probably similar. For fish, whole grains, and legumes, more recent but smaller and less comprehensive meta-analyses were omitted from our data (9-11). These also provided similar effect estimates to the estimates we used. For some food groups, such as dairy products, fruits, and vegetables, systematic reviews of meta-analyses were available and supported the selection of the data sources (12, 13). For some food groups, such as white meat, meta-analyses with dose-response data were not found, and we needed to make assumptions based on which yields more uncertainty in model output. Many of the background studies were adjusted for other food groups. It can be argued that food groups are interrelated and thus not independent. Studies presenting outcome measures with and without adjustment for other food groups have generally indicated minimal changes in the outcome measures (14-16). To account for this possibility, we added sensitivity analyses model adjustment.

To avoid reporting estimates for insufficiently studied and unsustainable diet alternatives, the model does not report estimates if the total energy consumption for the diet input was below 4 000 kJ/day or above 16 000 kJ/day. Energy estimates per food group were obtained from a food content database (17). The energy estimates were 8935 kJ/day for Western diets, 8275 kJ/day for feasible-approach diets, 7875 kJ/day for optimal vegetarian/vegan diets, and 7615 kJ/day for optimal diets. The effect of energy restriction on longevity was not considered.

**Current diets in various countries**

We extracted data on current food consumption in females and males in Nordic and Baltic countries including Norway, Sweden, Denmark, Iceland, Finland, Estonia, Lithuania, and Latvia, see details in Supplementary Text S2, Table 1. The energy estimates ranged from 4860 kJ/day for Lithuanian females to 9409 kJ/day for Norwegian males, Table 1.

**Comparison diets**

We compared current national dietary patterns to NNR diet. Intake levels and examples for foods for NNR diet are given below (rounded off, in fresh-weight/ready-to-eat, except for refined grains):

- Whole grains (fresh weight): 300 g (e.g., 2 thin slices of rye bread and one small bowl of whole grain cereal, and some whole grain rice). For whole grains, 225g of fresh weight corresponds to about 75g dry weight, equivalent of 7 servings/day).
- Fruits and vegetables recommended intake is 500-800 g/d. We divided this between these 2 categories and added 100 g/d of potatoes to the vegetables group.
  - Vegetables: 400g (five servings, e.g., one big tomato, one sweet pepper, mixed salad leaves, a half avocado, and a small bowl of vegetable soup)
  - Fruits: 300g (five servings, e.g., one apple, banana, orange, kiwi, and a handful of berries)
- Legumes intake should be a significant part of daily meals. We estimated the recommended intake to be 50-150 g/d.
- Nuts recommended intake: 20-30 g/d
- Fish: 300-450 g/week (40-65 g/d) (e.g., one big slice of herring)
- Eggs: moderate intake, 0.5- 1 egg/d equal to 30g/d
- Milk/dairy: 400g (e.g., one cup of yogurt)
- Refined grains (dry weight): unchanged (e.g., refined grains in bread if mixed whole/refined bread)
- Red meat (unprocessed and processed meat): 350 g/week max, limit processed meat as much as possible. We estimated this recommendation to 40 g/d of red meat and 10 g/d of processed meat for a feasible diet and no intake of red meat for a full-potential interpretation of NNR 2023.
- White meat should not be increased from the default diet which was 25-50 g/d.
- Sugar-sweetened beverages should be limited (no intake).
- Added sugar should be limited (no intake)
- The uncategorized proportion was estimated based on remaining energy consumption after considering every food group.

**Energy calculation**

To calculate energy in kcal, we use energy expenditure prediction equations suggested by Heymsfield et al. (18):

1. Male: Total estimated energy (kcal/day) is 864- 9.72 * age + activity coefficient * (14.2 * weight (kg) + 503 * height (m))
2. Females: Total estimated energy (kcal/day) is 387-7.31 * age + activity coefficient * (10.9 * weight (kg) + 660.7 * height (m))

To calculate energy in kJ/day, the estimates in kcal/day were multiplied with 4.184. The energy default for the reference persons were 2171 kcal/day.

**Online calculator**

The defaults on the left food panel, representing the pre-change diet, are established based on current dietary patterns observed in the corresponding countries. The food panel on the right illustrates the dietary modifications that have been made. Upon selecting the "New diet: NNR 2023" option, the sliders located in the right panel are automatically modified to align with the dietary patterns prescribed by the NNR guidelines.

**Hazard ratios extracted from meta-analyses**

Hazard ratios (with uncertainty intervals) for various food groups with uncertainty limits (orange/red labels). Reference intake and energy levels per food groups are also indicated.

Supplementary Figure 1. Hazard ratios extracted from meta-analyses

**Evidence**

To assess the quality of evidence for each food group from the meta-analyses, we use NutriGrade, a version of GRADE adapted to nutritional studies (19). Certainty of evidence is categorized as “very low” (0-3.99), “low” (4-5.99), “moderate” (6-7.99), or “high” (8-10). The quality of evidence was “high” for whole grains (NutriGrade score: 8), “moderate” for fish (7.75), processed meat (7.5), nuts (7), red meat (6.5), legumes (6), and dairy (6), “low” for vegetables (5.8), fruits (5.8), SSBs (5.5), and refined grains (5), and “very low” for eggs (3.8) and white meat (2). We further constructed an overall quality score by taking the mean of the NutriGrade scores for each of the food groups weighted by their absolute contribution to life expectancy. The quality of the meta-analyses was assessed with the AMSTAR-2 tool (20). The quality of the meta-analyses was rated as high for studies on all included meta-analyses (2-5, 7), except for the meta-analysis on white meat that was rated as moderate (6). Moreover, the potential long-term health implications resulting from the excessive consumption of food containing elevated levels of toxins, such as dioxins and polychlorinated biphenyls, which are particularly pertinent to some varieties of fish and seafood, have not been taken into account (21, 22).

**Uncertainty intervals**

To calculate 95% uncertainty intervals (UI) for the overall, and food-specific, associations between dietary change with life expectancy, we drew a number at random within the 95% confidence interval for each food group and used this as the model input. This procedure was repeated 200 times and 95% uncertainty limits were selected as the 2.5- and 97.5 percentiles of the distribution of the 200 replicates (with a fixed seed as starting point).

We also added uncertainty from uncategorized food items, with uncertainty corresponding to replacement with added oils (that had neutral mid estimates). The energy contribution of this “replacement”, was balanced to provide typical energy needs for the reference persons.

**Correlation and model adjustments**

Intakes of food groups correlate with the intakes of other food groups. Although risk estimates for each food group for many of the studies were adjusted for some other food groups, many might not have fully accounted for this correlation. For the analyses on which the hazard ratios are based do not account fully for this correlation, the hazard ratios may be biased away from the null. Therefore, as explained in Fadnes et al. (2022), our model features a parameter, m, ranging from 0 to 1.0, where m=1 means that hazard ratios for all food groups are assumed independent after the adjustments summarized in the meta-analysis, and m=0.5 means that hazard ratios will be moved half-way towards 1 (the null association is hazard ratio of 1).

Assuming first that HR_0_<1, we calculated alternative hazard ratios (HR_a_) based on HR_0_, the hazard ratio from the meta-analyses for a given change of intake for a given food group:

HR_a_ = HR_0_ + (1- HR_0_)*(1-m) ,

where m is a parameter taking on values from 0.5 to 1.0. If 0.5<m<1, the model becomes more conservative in the sense that the effect of dietary changes is reduced.

The core-adjusted hazard ratios for mortality for each food group were adjusted for age, sex, socio-demographic area, smoking, alcohol consumption, and activity level. The mutually adjusted hazard ratios were adjusted additionally for other important food groups (fruits, vegetables, nuts, red- and processed meats). The mutually adjusted hazard ratios corresponded to core-adjusted hazard ratios with an m ranging from 0.83 to 0.88. To account for the correlation between all the included food group categories, core-adjusted hazard ratios with an m=0.75 were used. We also present sensitivity analyses for m=0.5 (conservative estimates), and m=1.0.

**Time to full effect**

We modified this approach by considering “change in diet” as a condition that may have both a positive and a negative health impact. Health gains from diet changes are generally linked to a reduction in cardiovascular disease, cancer, and diabetes mortality (2-5), all among the leading causes of mortality globally (23). It has earlier been assumed that reversing the process of cardiovascular disease following reductions in major cardiovascular risk factors would require decades, but it has later been argued that cardiovascular disease mortality can change to some degree even within a few years (24, 25). For cancers, the time perspective is likely to be longer. It has been indicated for associations between fruit and vegetable consumption and the risk of lung cancer that associations for studies with more than 10 years of follow-up on fruits and vegetables are stronger than those with less than 10 years (26). More evidence on the time perspective is available for risk factors such as tobacco, where meta-analyses for duration of smoking have indicated that associations between duration of tobacco smoking and risk of lung cancer is substantially higher with 50 years of smoking than 20 years of smoking (27). To account for the expected time delay between dietary change and the development of diseases such as cardiovascular disease and cancer (24-26), while weighting in the morbidity burden of these (2-5, 23), we assumed that time to full effect was 20 years with an inverse S-shaped decrease in mortality (S Fig170). We also conducted sensitivity analyses with delays of 5 years to 50 years.

**Patient involvement**

No patients were involved in setting the research question or the outcome measures, nor were they involved in developing plans for design or implementation of the study. No patients were asked to advise on interpretation or writing up of results. There are no plans to disseminate the results of the research to study participants or the relevant patient community.

**Ethics**

This study exclusively utilized publicly accessible data sources, hence obviating the need for ethical clearance. The authors of this study followed the guidelines outlined in the Transparent Reporting of a Multivariable Prediction Model for Individual Prognosis or Diagnosis (TRIPOD) to ensure the transparency of their reporting (28).

**Medline/PubMed search to estimate number of nutritional articles per year**

(("Nutritional Sciences"[Mesh] OR "Nutritional Status"[Mesh] OR "Child Nutrition Sciences"[Mesh] OR "Parenteral Nutrition Solutions"[Mesh] OR "Nutritional Physiological Phenomena"[Mesh] OR "Diet, Food, and Nutrition"[Mesh] OR "Enteral Nutrition"[Mesh] OR "Infant Nutrition Disorders"[Mesh] OR "Nutrition Disorders"[Mesh] OR "Nutrition Surveys"[Mesh] OR "Parenteral Nutrition"[Mesh] OR "Parenteral Nutrition, Total"[Mesh] OR "Child Nutrition Disorders"[Mesh] OR "Nutrition Assessment"[Mesh] OR "Parenteral Nutrition, Home"[Mesh] OR "Parenteral Nutrition, Home Total"[Mesh] OR "Animal Nutrition Sciences"[Mesh] OR "Fetal Nutrition Disorders"[Mesh] OR "Nutrition Policy"[Mesh] OR "Nutrition Therapy"[Mesh]) OR ( "Elder Nutritional Physiological Phenomena"[Mesh] OR "Sports Nutritional Physiological Phenomena"[Mesh] OR "Sports Nutritional Sciences"[Mesh] OR "Prenatal Nutritional Physiological Phenomena"[Mesh] OR "Nutritive Value"[Mesh] OR "Nutritional Requirements"[Mesh] OR "Animal Nutritional Physiological Phenomena"[Mesh] OR "Maternal Nutritional Physiological Phenomena"[Mesh] OR "Adolescent Nutritional Physiological Phenomena"[Mesh] OR "Infant Nutritional Physiological Phenomena"[Mesh] OR "Child Nutritional Physiological Phenomena"[Mesh] OR "Food Assistance"[Mesh] OR "Food Labeling"[Mesh] OR "Parenteral Nutrition Solutions" [Pharmacological Action] )) AND ("2019/01/01"[Date - Publication] : "2019/12/31"[Date - Publication])

Last part with year is changed for each analysis

- 2022 (by 2022-12-06): 60,194 results.
- 2021: 77,896 results.
- 2020: 80,264 results.
- 2019: 75,760 results.
- 2018: 71,860 results.
- 2017: 68,485 results.
- Total (by 2022-12-06): 1,587,841 results

# String used in PubMed to identify meta-analyses for setting hazard ratios

(grains[title/abstract] OR fruits[title/abstract] OR vegetables[title/abstract] OR nuts[title/abstract] OR legumes[title/abstract] OR fish[title/abstract] OR eggs[title/abstract] OR milk[title/abstract] OR dairy[title/abstract] OR meat*[title/abstract] OR "sugar-sweetened beverages"[title/abstract]) AND mortality[title/abstract] AND Meta-Analysis[filter].

The search was conducted 26^th^ of April 2021 and 222 references were evaluated.

**Supplementary Text 2: Estimated dietary intake of various countries**

We extracted data on current food consumption in Nordic and Baltic countries. We divided the main food groups into these categories (grams per day): Refined grains, whole grain products, vegetables, fruits, legumes, nuts, fish, white meat, red meat, egg, milk and dairy products, added oil, sugar-sweetened beverages, and added sugar. We extract the default diet of Nordic and Baltic countries mainly from the recently published articles by Lemming and Pitsi (29) while checking the original reports used in that study to fit better with our definition of the food groups. Whole grains consumption was mostly reported in dry weight, and we converted them into fresh weight using the estimated ratio of 1g whole grain (dry weight) = 3.33 g whole grain product (fresh weight). Legumes consumption was mostly reported as dry weight, we estimated 1 g of dry legumes corresponds to 2.5 g of fresh weight. Vegetables also include the consumption of potatoes. The milk and dairy group includes daily consumption of milk and estimated milk in cheese (cheese (g/d) *7.5).

# Denmark

Danskernes kostvaner, a national survey conducted in Denmark in 2011-2013 was used to extract food patterns (30). This survey used a 7-day record method with 3016 participants aged 18-75 years old. Since red meat and processed meat were not reported separately, we estimated their consumption based on Norway’s consumption.

# Finland

We gathered information about Finland's dietary preferences from a national study FINDIET performed in 2017 (31, 32). This survey used a 2*24 recall method with 3099 participants aged 25-75. Whole grain consumption was extracted from FinRavinto report in 2017 (32).

# Iceland

We extracted data on food patterns in Iceland from a national survey “Hvað borða ĺslendingar?” conducted in 2019-21(33). This survey used a 2*24 recall method with 1312 participants aged 18-80 years old. The intake of refined grains was not reported in the most recent report, so we used the data from the previous survey in 2010-11(34).

# Norway

We extracted data on food patterns in Norway from a national survey Norkost3 conducted in 2010-11 (35). Norkost 3 used a 2*24 hour recall method on 1787 participants aged 18-70 years old. Red meat and processed meat consumption were extracted from a report in 2013 titled “Nutritional evaluation of lowering consumption of meat and meat products in the Nordic context”(36).

# Estonia

We used Estonian National Dietary Survey 2014 to extract estimates of each food group's consumption (37). This survey used a 2*24 hour recall method with 2713 participants aged 18-74 years old.

# Latvia

We extracted estimates of food groups from Latvian National Dietary Survey on the general population conducted in 2007-09 (38). This survey used a 2*24-hour recall method with 1377 participants aged 17-64 years old.

# Lithuania

We extracted the data from the study of actual nutrition and nutrition habits of Lithuanian adult population conducted in 2013-2014 (39). This survey used a 2*24 hour recall method with 2513 participants aged 19-75 years old. The consumption of meat was reported as an overall number for red meat, processed meat, and white meat. We estimated the consumption of subgroups of meat food groups based on Latvia’s consumption patterns.

# Sweden

We used Riksmaten vuxna national survey to estimate the daily intake of food groups (40). This survey was conducted in 2010-11, with 1797 participants aged 18-80, using a 4-day record method. Processed meat and red meat consumption was extracted from a report in 2014 titled “Consumption of red meat and charcuterie products and association with colon and rectal cancer”(41).

# References

1. The Institute for Health Metrics and Evaluation (IHME). The Global Health Data Exchange (GHDx) Institute for Health Metrics and Evaluation, University of Washington, ; 2020 [Available from: <http://ghdx.healthdata.org/gbd-results-tool>.

2. Aune D, Giovannucci E, Boffetta P, Fadnes LT, Keum N, Norat T, et al. Fruit and vegetable intake and the risk of cardiovascular disease, total cancer and all-cause mortality-a systematic review and dose-response meta-analysis of prospective studies. Int J Epidemiol. 2017;46(3):1029-56.

3. Aune D, Keum N, Giovannucci E, Fadnes LT, Boffetta P, Greenwood DC, et al. Whole grain consumption and risk of cardiovascular disease, cancer, and all cause and cause specific mortality: systematic review and dose-response meta-analysis of prospective studies. BMJ. 2016;353:i2716.

4. Aune D, Keum N, Giovannucci E, Fadnes LT, Boffetta P, Greenwood DC, et al. Nut consumption and risk of cardiovascular disease, total cancer, all-cause and cause-specific mortality: a systematic review and dose-response meta-analysis of prospective studies. BMC Med. 2016;14(1):207.

5. Schwingshackl L, Schwedhelm C, Hoffmann G, Lampousi AM, Knüppel S, Iqbal K, et al. Food groups and risk of all-cause mortality: a systematic review and meta-analysis of prospective studies. Am J Clin Nutr. 2017;105(6):1462-73.

6. Abete I, Romaguera D, Vieira AR, Lopez de Munain A, Norat T. Association between total, processed, red and white meat consumption and all-cause, CVD and IHD mortality: a meta-analysis of cohort studies. Br J Nutr. 2014;112(5):762-75.

7. Abdelhamid AS, Martin N, Bridges C, Brainard JS, Wang X, Brown TJ, et al. Polyunsaturated fatty acids for the primary and secondary prevention of cardiovascular disease. Cochrane Database Syst Rev. 2018;7:CD012345.

8. Zeraatkar D, Han MA, Guyatt GH, Vernooij RWM, El Dib R, Cheung K, et al. Red and Processed Meat Consumption and Risk for All-Cause Mortality and Cardiometabolic Outcomes: A Systematic Review and Meta-analysis of Cohort Studies. Ann Intern Med. 2019.

9. Wan Y, Zheng J, Wang F, Li D. Fish, long chain omega-3 polyunsaturated fatty acids consumption, and risk of all-cause mortality: a systematic review and dose-response meta-analysis from 23 independent prospective cohort studies. Asia Pac J Clin Nutr. 2017;26(5):939-56.

10. Zhang B, Zhao Q, Guo W, Bao W, Wang X. Association of whole grain intake with all-cause, cardiovascular, and cancer mortality: a systematic review and dose-response meta-analysis from prospective cohort studies. European journal of clinical nutrition. 2018;72(1):57-65.

11. Li H, Li J, Shen Y, Wang J, Zhou D. Legume Consumption and All-Cause and Cardiovascular Disease Mortality. Biomed Res Int. 2017;2017:8450618.

12. Yip CSC, Chan W, Fielding R. The Associations of Fruit and Vegetable Intakes with Burden of Diseases: A Systematic Review of Meta-Analyses. Journal of the Academy of Nutrition and Dietetics. 2019;119(3):464-81.

13. Cavero-Redondo I, Alvarez-Bueno C, Sotos-Prieto M, Gil A, Martinez-Vizcaino V, Ruiz JR. Milk and Dairy Product Consumption and Risk of Mortality: An Overview of Systematic Reviews and Meta-Analyses. Advances in nutrition. 2019;10(suppl_2):S97-s104.

14. Roswall N, Sandin S, Lof M, Skeie G, Olsen A, Adami HO, Weiderpass E. Adherence to the healthy Nordic food index and total and cause-specific mortality among Swedish women. Eur J Epidemiol. 2015;30(6):509-17.

15. Vormund K, Braun J, Rohrmann S, Bopp M, Ballmer P, Faeh D. Mediterranean diet and mortality in Switzerland: an alpine paradox? Eur J Nutr. 2015;54(1):139-48.

16. Prinelli F, Yannakoulia M, Anastasiou CA, Adorni F, Di Santo SG, Musicco M, et al. Mediterranean diet and other lifestyle factors in relation to 20-year all-cause mortality: a cohort study in an Italian population. Br J Nutr. 2015;113(6):1003-11.

17. Mattilsynet. Matvaretabellen [Available from: <https://www.matvaretabellen.no/?language=en>.

18. Heymsfield SB, Harp JB, Rowell PN, Nguyen AM, Pietrobelli A. How much may I eat? Calorie estimates based upon energy expenditure prediction equations. Obes Rev. 2006;7(4):361-70.

19. Schwingshackl L, Knuppel S, Schwedhelm C, Hoffmann G, Missbach B, Stelmach-Mardas M, et al. Perspective: NutriGrade: A Scoring System to Assess and Judge the Meta-Evidence of Randomized Controlled Trials and Cohort Studies in Nutrition Research. Advances in Nutrition. 2016;7(6):994-1004.

20. Shea BJ, Reeves BC, Wells G, Thuku M, Hamel C, Moran J, et al. AMSTAR 2: a critical appraisal tool for systematic reviews that include randomised or non-randomised studies of healthcare interventions, or both. BMJ. 2017;358:j4008.

21. Malisch R, Kotz A. Dioxins and PCBs in feed and food--review from European perspective. Sci Total Environ. 2014;491-492:2-10.

22. Faroon O, Jones D, de Rosa C. Effects of polychlorinated biphenyls on the nervous system. Toxicol Ind Health. 2000;16(7-8):305-33.

23. Collaborators GBDCoD. Global, regional, and national age-sex-specific mortality for 282 causes of death in 195 countries and territories, 1980-2017: a systematic analysis for the Global Burden of Disease Study 2017. Lancet. 2018;392(10159):1736-88.

24. Capewell S, O'Flaherty M. Can dietary changes rapidly decrease cardiovascular mortality rates? Eur Heart J. 2011;32(10):1187-9.

25. Capewell S, O'Flaherty M. Rapid mortality falls after risk-factor changes in populations. Lancet. 2011;378(9793):752-3.

26. Wang Y, Li F, Wang Z, Qiu T, Shen Y, Wang M. Fruit and vegetable consumption and risk of lung cancer: a dose-response meta-analysis of prospective cohort studies. Lung Cancer. 2015;88(2):124-30.

27. Lee PN, Forey BA, Coombs KJ. Systematic review with meta-analysis of the epidemiological evidence in the 1900s relating smoking to lung cancer. BMC Cancer. 2012;12:385.

28. Collins GS, Reitsma JB, Altman DG, Moons KG. Transparent reporting of a multivariable prediction model for individual prognosis or diagnosis (TRIPOD): the TRIPOD Statement. BMC Med. 2015;13:1.

29. Lemming EW, Pitsi T. The Nordic Nutrition Recommendations 2022 - food consumption and nutrient intake in the adult population of the Nordic and Baltic countries. Food Nutr Res. 2022;66.

30. Pedersen AN, Christensen T, Matthiessen J, Knudsen VK, Sørensen MR, Biltoft-Jensen AP, et al. Danskernes kostvaner 2011-2013: DTU Fødevareinstituttet, Danmarks Tekniske Universitet; 2015.

31. Kaartinen N, Tapanainen H, Reinivuo H, Pakkala H, Aalto S, Raulio S, et al. The Finnish National Dietary Survey in Adults and Elderly (FinDiet 2017). EFSA Supporting Publications. 2020;17(8):1914E.

32. Valsta L, Kaartinen N, Tapanainen H, Männistö S, Sääksjärvi K. Ravitsemus Suomessa: FinRavinto 2017-tutkimus. 2018.

33. Gunnarsdottir S, Gudmannsdottir R, Thorgeirsdottir H, Torfadottir JE, Steingrimsdottir L, Tryggvadottir EA, et al. Hvað borða Íslendingar? Könnun á mataræði Íslendinga 2019-2021 (What do Icelanders eat? Survey of the diet of Icelanders 2019-2021). Reykjavik: Directorate of Health/Unit for Nutrition Research, University of Iceland; 2022.

34. Steingrímsdóttir L, Þorgeirsdóttir H, Ólafsdóttir AS. Hvað borða íslendingar. Könnun á mataræði Íslendinga. 2002.

35. Totland TH, Melnæs BK, Lundberg-Hallen N, Helland-Kigen KM, Lund-Blix NA, Myhre JB, et al. Norkost 3 En landsomfattende kostholdsundersøkelse blant menn og kvinner i Norge i alderen 18-70 år, 2010-11. Oslo: Helsedirektoratet. 2012;67.

36. Tetens I, Hoppe C, Frost Andersen L, Helldán A, Warensjö Lemming E, Trolle E, et al. Nutritional evaluation of lowering consumption of meat and meat products in the Nordic context. København: Nordisk ministerråd; 2013 2013. 88 p.

37. Development NIfH, Nurk E, Nelis K, Saamel M, Martverk M, Nelis L. National Dietary Survey among 11-74 years old individuals in Estonia. EFSA Supporting Publications. 2017;14(4):1198E.

38. Institute of Food Safety AH, BIOR E, Siksna I, Valciņa O, Ozoliņš G, Goldmanis M. Latvian National Dietary Survey on the general population. EFSA Supporting Publications. 2017;14(11):1307E.

39. Dobrovolskij V, Stukas R. Lietuvos gyventojų mitybos įpročiai 2013 metais. Sveikatos mokslai. 2013;23(4):34-41.

40. Amcoff E. Riksmaten-vuxna 2010-11: Livsmedels-och näringsintag bland vuxna i Sverige: Livsmedelsverket; 2012.

41. Farvid MS, Sidahmed E, Spence ND, Mante Angua K, Rosner BA, Barnett JB. Consumption of red meat and processed meat and cancer incidence: a systematic review and meta-analysis of prospective studies. Eur J Epidemiol. 2021;36(9):937-51.

| Country | Sex | NNR2023 Diet | Whole grain | Vegetable | Fruit | Nuts | Legumes | Fish | Egg | Milk | Red meat | Processed meat | SSB | Added sugar | Total** |
| --- | --- | --- | --- | --- | --- | --- | --- | --- | --- | --- | --- | --- | --- | --- | --- |
| Denmark | F | Feasible | 0.8 | 0.0 | 0.0 | 1.1 | 0.3 | 0.0 | -0.4 | 0.2 | 0.7 | 0.7 | 0.3 | 0.1 | 3.2 |
|  |  | Full-potential | 0.8 | 0.1 | 0.2 | 1.0 | 1.0 | 0.1 | 0.0 | 0.4 | 0.7 | 0.8 | 0.3 | 0.6 | 5.9 |
|  | M | Feasible | 0.9 | 0.1 | 0.1 | 1.1 | 0.3 | 0.0 | -0.4 | 0.1 | 0.2 | 0.5 | 0.3 | 0.1 | 3.4 |
|  |  | Full-potential | 0.9 | 0.2 | 0.2 | 1.1 | 1.0 | 0.1 | 0.0 | 0.2 | 0.7 | 0.8 | 0.3 | 0.6 | 6.3 |
| Estonia | F | Feasible | 0.7 | 0.2 | 0.0 | 1.0 | 0.3 | 0.1 | -0.4 | -0.2 | 0.0 | 0.0 | 0.1 | 0.3 | 2.1 |
|  |  | Full-potential | 0.7 | 0.2 | 0.1 | 1.0 | 1.0 | 0.2 | 0.0 | 0.0 | 0.4 | 0.3 | 0.1 | 0.7 | 4.8 |
|  | M | Feasible | 1.0 | 0.3 | 0.0 | 1.1 | 0.3 | 0.1 | -0.4 | -0.2 | 0.0 | 0.0 | 0.1 | 0.3 | 2.6 |
|  |  | Full-potential | 1.0 | 0.3 | 0.2 | 1.1 | 1.1 | 0.2 | 0.0 | 0.0 | 0.4 | 0.3 | 0.1 | 0.7 | 5.7 |
| Finland | F | Feasible | 0.4 | 0.2 | 0.1 | 0.5 | 0.2 | 0.1 | -0.4 | 0.2 | 0.0 | 0.3 | 0.1 | 0.1 | 1.8 |
|  |  | Full-potential | 0.4 | 0.2 | 0.3 | 0.5 | 0.9 | 0.2 | 0.0 | 0.3 | 0.4 | 0.6 | 0.1 | 0.5 | 4.4 |
|  | M | Feasible | 0.5 | 0.2 | 0.2 | 0.6 | 0.3 | 0.1 | -0.4 | 0.1 | 0.0 | 0.3 | 0.1 | 0.2 | 2.1 |
|  |  | Full-potential | 0.5 | 0.3 | 0.3 | 0.6 | 1.0 | 0.2 | 0.0 | 0.3 | 0.4 | 0.6 | 0.1 | 0.6 | 5.0 |
| Iceland | F | Feasible | 0.2 | 0.4 | 0.4 | 1.0 | 0.2 | 0.0 | -0.4 | 0.1 | 0.0 | 0.3 | 0.1 | 0.1 | 2.6 |
|  |  | Full-potential | 0.2 | 0.5 | 0.5 | 1.0 | 0.9 | 0.1 | 0.1 | 0.2 | 0.4 | 0.6 | 0.1 | 0.6 | 5.1 |
|  | M | Feasible | 0.4 | 0.5 | 0.4 | 1.1 | 0.2 | 0.0 | -0.4 | 0.0 | 0.0 | 0.3 | 0.1 | 0.1 | 2.8 |
|  |  | Full-potential | 0.4 | 0.6 | 0.6 | 1.1 | 0.9 | 0.1 | 0.1 | 0.2 | 0.4 | 0.6 | 0.1 | 0.6 | 5.6 |
| Latvia | F | Feasible | 0.7 | 0.1 | 0.2 | 0.5 | 0.2 | 0.1 | -0.4 | -0.3 | 0.0 | 0.8 | 0.1 | 0.5 | 2.6 |
|  |  | Full-potential | 0.7 | 0.1 | 0.3 | 0.5 | 0.9 | 0.2 | 0.0 | -0.1 | 0.4 | 1.1 | 0.1 | 0.9 | 5.4 |
|  | M | Feasible | 1.0 | 0.2 | 0.3 | 0.6 | 0.3 | 0.1 | -0.5 | -0.3 | 0.0 | 0.9 | 0.1 | 0.4 | 3.3 |
|  |  | Full-potential | 1.0 | 0.2 | 0.4 | 0.6 | 1.0 | 0.2 | 0.0 | -0.1 | 0.5 | 1.3 | 0.1 | 0.9 | 6.5 |
| Lithuania | F | Feasible | 1.0 | 0.2 | 0.1 | 1.0 | 0.3 | 0.1 | -0.4 | -0.2 | -0.1 | 0.7 | 0.1 | 0.5 | 3.4 |
|  |  | Full-potential | 1.0 | 0.3 | 0.2 | 0.9 | 0.9 | 0.2 | 0.0 | 0.0 | 0.3 | 1.0 | 0.1 | 0.9 | 6.1 |
|  | M | Feasible | 1.2 | 0.4 | 0.2 | 1.2 | 0.3 | 0.1 | -0.5 | -0.2 | -0.1 | 0.8 | 0.1 | 0.4 | 4.1 |
|  |  | Full-potential | 1.2 | 0.5 | 0.4 | 1.2 | 1.1 | 0.2 | 0.0 | 0.0 | 0.3 | 1.1 | 0.1 | 0.9 | 7.3 |
| Norway | F | Feasible | 0.3 | 0.2 | 0.1 | 1.0 | 0.3 | -0.1 | -0.4 | 0.1 | 0.1 | 0.5 | 0.2 | 0.1 | 2.5 |
|  |  | Full-potential | 0.3 | 0.3 | 0.2 | 1.0 | 1.0 | 0.0 | 0.0 | 0.3 | 0.5 | 0.8 | 0.2 | 0.5 | 5.1 |
|  | M | Feasible | 0.4 | 0.3 | 0.2 | 1.0 | 0.3 | -0.1 | -0.4 | 0.0 | 0.1 | 0.5 | 0.2 | 0.1 | 2.8 |
|  |  | Full-potential | 0.4 | 0.3 | 0.3 | 1.0 | 1.0 | 0.0 | 0.0 | 0.2 | 0.5 | 0.8 | 0.2 | 0.5 | 5.5 |
| Sweden | F | Feasible | 0.6 | 0.1 | 0.2 | 1.0 | 0.2 | 0.0 | -0.3 | -0.1 | 0.1 | 0.3 | 0.1 | 0.3 | 2.5 |
|  |  | Full-potential | 0.6 | 0.2 | 0.3 | 0.9 | 0.8 | 0.1 | 0.1 | 0.1 | 0.5 | 0.6 | 0.1 | 0.7 | 5.1 |
|  | M | Feasible | 0.7 | 0.2 | 0.2 | 1.0 | 0.2 | 0.0 | -0.3 | -0.2 | 0.1 | 0.3 | 0.1 | 0.3 | 2.6 |
|  |  | Full-potential | 0.7 | 0.2 | 0.4 | 1.0 | 0.8 | 0.1 | 0.1 | 0.0 | 0.5 | 0.6 | 0.1 | 0.7 | 5.4 |

**Supplementary Table 1**. Estimated food-specific life expectancy changes in years from sustained change from current diets in the Nordic and Baltic countries to feasible- and full-potential interpretation of the NNR2023* among 40-year-old females (F) and males (M).

*Refined grains and white meat had no contributions since the new diet includes no change in the intake. ** The total sum of the contributions is less than the estimated total LE gain because of the estimated contribution of uncategorized group.

**Supplementary Table 2**. Estimated percentage of food-specific life expectancy changes in years from sustained change from current diets in the Nordic and Baltic countries to feasible- and full-potential interpretation of the NNR2023* among 40-year-old females (F) and males (M).

| Country | Sex | NNR2023 | Whole grain | Vegetable | Fruit | Nuts | Legumes | Fish | Egg | Milk | Red meat | Processed meat | SSB | Added sugar |
| --- | --- | --- | --- | --- | --- | --- | --- | --- | --- | --- | --- | --- | --- | --- |
| Denmark | F | Feasible | 19.9 | 1.0 | 1.0 | 27.4 | 8.0 | 0.0 | -10.9 | 4.4 | 18.9 | 18.1 | 8.5 | 3.6 |
|  |  | Full-potential | 13.3 | 1.2 | 3.1 | 17.1 | 17.1 | 1.9 | 0.0 | 6.0 | 11.2 | 13.8 | 5.7 | 9.5 |
|  | M | Feasible | 27.1 | 3.7 | 2.2 | 32.6 | 9.5 | 0.0 | -12.9 | 1.8 | 7.4 | 15.4 | 8.9 | 4.3 |
|  |  | Full-potential | 14.7 | 2.7 | 3.5 | 17.8 | 16.6 | 1.8 | 0.0 | 4.0 | 11.1 | 13.6 | 4.9 | 9.4 |
| Estonia | F | Feasible | 35.3 | 9.2 | 0.0 | 48.8 | 14.5 | 3.4 | -19.3 | -7.7 | 0.0 | 0.0 | 3.4 | 12.6 |
|  |  | Full-potential | 15.9 | 5.0 | 3.1 | 20.7 | 20.7 | 3.9 | 0.0 | 0.2 | 8.5 | 6.1 | 1.5 | 14.4 |
|  | M | Feasible | 37.7 | 10.7 | 1.6 | 44.8 | 13.1 | 3.2 | -17.5 | -7.9 | 0.0 | 0.0 | 2.8 | 11.5 |
|  |  | Full-potential | 17.6 | 5.8 | 3.5 | 21.0 | 19.5 | 3.7 | 0.0 | -0.2 | 8.2 | 5.9 | 1.3 | 13.7 |
| Finland | F | Feasible | 21.5 | 9.6 | 6.2 | 29.9 | 13.0 | 4.5 | -22.6 | 9.0 | 0.0 | 15.3 | 6.2 | 7.3 |
|  |  | Full-potential | 8.8 | 4.9 | 5.8 | 10.7 | 20.4 | 4.2 | 0.0 | 7.9 | 9.3 | 13.0 | 2.6 | 12.5 |
|  | M | Feasible | 23.8 | 11.9 | 10.4 | 28.2 | 12.4 | 4.0 | -21.8 | 3.0 | 0.0 | 14.9 | 5.9 | 7.4 |
|  |  | Full-potential | 9.9 | 5.2 | 6.4 | 11.8 | 19.7 | 4.1 | 0.0 | 6.4 | 9.1 | 12.8 | 2.5 | 12.0 |
| Iceland | F | Feasible | 8.9 | 17.1 | 14.4 | 40.5 | 8.9 | 0.0 | -13.6 | 2.3 | 0.0 | 10.9 | 5.1 | 5.4 |
|  |  | Full-potential | 4.5 | 9.3 | 9.9 | 18.8 | 17.3 | 2.1 | 1.2 | 4.7 | 8.0 | 11.1 | 2.5 | 10.7 |
|  | M | Feasible | 14.1 | 18.5 | 15.2 | 38.8 | 8.7 | 0.0 | -13.0 | -1.4 | 0.0 | 10.1 | 4.0 | 5.1 |
|  |  | Full-potential | 7.1 | 10.0 | 10.2 | 19.5 | 16.8 | 2.0 | 1.1 | 2.7 | 7.7 | 10.7 | 2.0 | 10.2 |
| Latvia | F | Feasible | 28.7 | 2.4 | 5.9 | 20.5 | 9.1 | 2.8 | -15.7 | -10.2 | 0.0 | 32.3 | 4.3 | 20.1 |
|  |  | Full-potential | 14.4 | 2.0 | 5.7 | 8.9 | 17.2 | 3.6 | 0.0 | -1.8 | 7.9 | 21.9 | 2.2 | 18.1 |
|  | M | Feasible | 31.1 | 5.1 | 8.7 | 19.2 | 8.3 | 2.9 | -14.7 | -8.7 | 0.0 | 30.1 | 3.8 | 14.1 |
|  |  | Full-potential | 16.0 | 3.3 | 6.9 | 9.9 | 16.3 | 3.5 | 0.0 | -1.3 | 7.4 | 21.1 | 2.0 | 14.9 |
| Lithuania | F | Feasible | 29.8 | 7.5 | 3.4 | 31.1 | 9.3 | 2.2 | -12.1 | -5.9 | -3.4 | 20.5 | 1.9 | 15.8 |
|  |  | Full-potential | 16.8 | 4.9 | 4.2 | 16.4 | 16.4 | 3.1 | 0.0 | -0.3 | 4.9 | 16.6 | 1.0 | 15.9 |
|  | M | Feasible | 31.4 | 10.5 | 5.6 | 29.3 | 8.7 | 2.0 | -11.7 | -5.4 | -3.1 | 19.6 | 1.8 | 11.2 |
|  |  | Full-potential | 17.9 | 6.6 | 5.4 | 16.8 | 15.6 | 3.1 | 0.0 | -0.1 | 4.7 | 16.0 | 1.0 | 13.1 |
| Norway | F | Feasible | 12.1 | 8.9 | 2.8 | 41.5 | 12.1 | -3.2 | -16.1 | 5.6 | 4.4 | 19.4 | 7.3 | 5.2 |
|  |  | Full-potential | 6.0 | 5.2 | 4.2 | 19.0 | 19.0 | 0.6 | 0.0 | 6.3 | 10.1 | 15.3 | 3.6 | 10.7 |
|  | M | Feasible | 16.6 | 11.7 | 5.7 | 38.5 | 11.3 | -3.0 | -15.1 | 1.5 | 4.2 | 18.1 | 5.7 | 4.9 |
|  |  | Full-potential | 8.4 | 6.5 | 5.5 | 19.4 | 18.1 | 0.6 | 0.0 | 4.0 | 9.7 | 14.9 | 2.9 | 10.1 |
| Sweden | F | Feasible | 24.4 | 5.8 | 7.9 | 41.7 | 6.2 | 0.0 | -14.0 | -3.7 | 4.5 | 11.2 | 5.4 | 10.7 |
|  |  | Full-potential | 12.0 | 3.7 | 6.7 | 19.1 | 16.0 | 2.2 | 1.2 | 1.8 | 10.1 | 11.2 | 2.6 | 13.4 |
|  | M | Feasible | 26.0 | 7.5 | 9.4 | 40.6 | 5.9 | 0.0 | -13.4 | -5.9 | 4.3 | 10.6 | 4.3 | 10.6 |
|  |  | Full-potential | 12.8 | 4.5 | 7.4 | 20.0 | 15.5 | 2.1 | 1.2 | 0.4 | 9.9 | 11.1 | 2.1 | 13.0 |

*Refined grains and white meat had no contributions since the new diet includes no change in the intake.

**Supplementary Table 3**. The mean contribution of food groups to life expectancy (LE) change in years and the percentage of their contribution for feasible and full-potential interpretation of NNR2023*

|  | Mean all | Mean feasible | Mean full-potential | % all | % feasible | % full-potential |
| --- | --- | --- | --- | --- | --- | --- |
| Whole grain | 0.70 | 0.67 | 0.67 | 17 | 24 | 12 |
| Vegetable | 0.23 | 0.23 | 0.27 | 6 | 8 | 5 |
| Fruit | 0.22 | 0.17 | 0.31 | 5 | 6 | 6 |
| Nuts | 1.01 | 0.93 | 0.89 | 25 | 34 | 17 |
| Legumes | 0.57 | 0.26 | 0.94 | 14 | 10 | 18 |
| Fish | 0.08 | 0.03 | 0.14 | 2 | 1 | 3 |
| Egg | -0.17 | -0.40 | 0.02 | -4 | -15 | 0 |
| Milk | 0.03 | -0.06 | 0.13 | 1 | -2 | 2 |
| Refined grains (dry) | 0.00 | 0.00 | 0.00 | 0 | 0 | 0 |
| Red meat | 0.30 | 0.07 | 0.46 | 7 | 3 | 9 |
| Processed meat | 0.57 | 0.44 | 0.73 | 14 | 16 | 14 |
| White meat | 0.00 | 0.00 | 0.00 | 0 | 0 | 0 |
| Sugar-sweetened beverages | 0.12 | 0.14 | 0.14 | 3 | 5 | 3 |
| Added oil | 0.00 | 0.00 | 0.00 | 0 | 0 | 0 |
| Added sugar | 0.52 | 0.26 | 0.67 | 13 | 9 | 13 |
| Total | 4.12 | 2.73 | 5.35 | 100 | 100 | 100 |

*Refined grains and white meat had no contributions since the new diet includes no change in the intake.
